# Between ‘Pioneers’ of the Cardiovascular Revolution and Its ‘Late Followers’: Mortality Changes in the Czech Republic and Poland Since 1968

**DOI:** 10.1007/s10680-017-9456-y

**Published:** 2017-11-30

**Authors:** Agnieszka Fihel, Marketa Pechholdová

**Affiliations:** 1 Université Paris Nanterre, Nanterre, France; 2 University of Warsaw, Warsaw, Poland; 3 University of Economics, Prague, Czech Republic; 4 INED, Paris, France

**Keywords:** Mortality, Life expectancy, Causes of death, The Czech Republic, Poland

## Abstract

After several decades of stagnation, mortality in most Central European countries started to decrease after 1989. The Czech Republic and Poland were the first former Communist countries in this region to experience a rapid and sustained increase in life expectancy. This study focuses on the trends in cause-of-death mortality that have contributed to the recent progress in these two countries. The analysis is based on the cause-of-death time series (1968–2013) reconstructed in accordance with the 10th ICD revision, which makes the data fully comparable over the full period under study. Actual trends in cause-specific mortality are presented, and age, sex and causes of death components of life expectancy changes are disentangled. In both countries, the reduction in cardiovascular mortality at adult and old ages was crucial for the increase in life expectancy after 1991. Results are discussed in the context of institutional changes that occurred after the fall of Communism, such as the reorientation of health policies and the emergence of non-governmental organizations. Changes in health-related attitudes and behaviours as well as structural changes in societies, notably the rising share of persons with tertiary education, are also discussed.

## Introduction

In the post-World War II period, European countries experienced changes in mortality levels in very different ways. While all European countries registered a steady rise in life expectancy levels, mainly as a result of an accelerated decline in infant mortality and infectious diseases, this rise stalled in the 1960s as new epidemiologic threats emerged in the form of increasing mortality from cardiovascular diseases, traffic accidents and other causes related to risky behaviours. According to the theory of *epidemiologic transition* (Omran 1971), these unfavourable phenomena were expected to hold back any further progress in life expectancy. Despite this claim, however, Western European societies appeared to have overcome these challenges in a relatively smooth fashion by implementing new medical technologies, comprehensive health policies and individual prevention measures (Vallin and Meslé 2006). In contrast, the Central and Eastern European countries experienced unfavourable trends until the 1990s (Meslé 1991; Vallin and Meslé 2001).

The Czech Republic and Poland were the first Communist countries to see the stagnation or, for some population groups, deterioration in life expectancy. Both countries were also the first^1^ former Communist countries in this region to see a sustained increase in life expectancy, and to this day they continue to be the most advanced in this process (Meslé 2004; Rychtaříková 2004). In this study, we want to gain a deeper understanding of the epidemiologic tendencies that contributed both to the unfavourable life expectancy changes in the Communist period and to the more recent progress in these two countries. To this end, we analyse the dynamics in cause-specific mortality and identify the most important causes of death that have underlain life expectancy changes. We study large cause-of-death categories that group entities into similar pathophysiologic and behavioural origins, for instance infectious diseases or smoking-related cancers. This is because our goal is to indicate that social and economic changes may have been related to the recent changes in cause-specific mortality and to recent improvements in life expectancy. We contrast the Czech Republic and Poland with two other countries representing different sequences of changes in their health situations: France, on the one hand, as a ‘pioneer’ in applying effective health policies and in neutralizing the major determinants of risky behavioural and societal patterns, and Russia, on the other hand, as a ‘late-comer’ where the nascent improvement in the health situation might still be fragile. In the discussion, we present three possible explanations for the advantageous mortality changes in the Czech Republic and Poland: successful reforms in the political and economic systems; changes in attitudes and behaviours regarding the main risk factors; and an important shift in the structure of education.

## Background

**Fig. 1:**
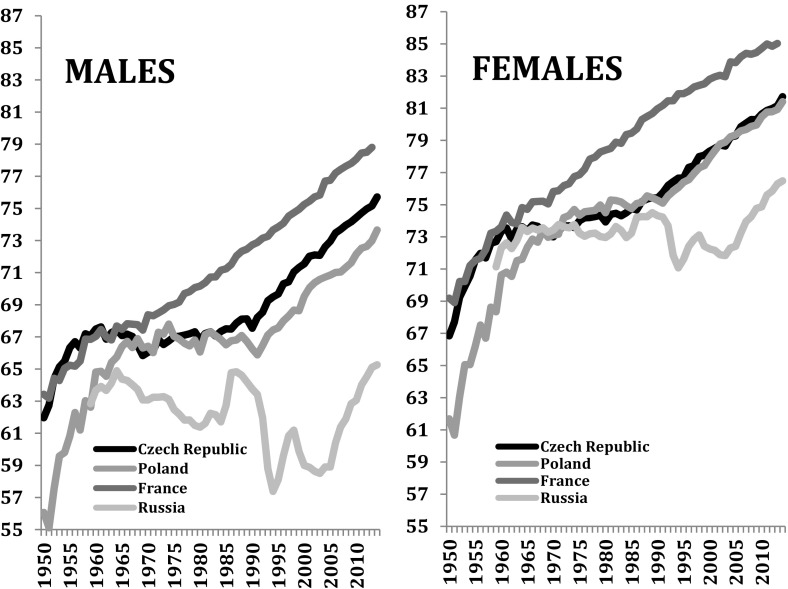
Life expectancy at birth in Czech Republic, France, Poland and Russia, 1950–2014. *Source*: Own elaboration based on Bolesławski (1996), Human Mortality Database (2015)

Just after World War II (WWII) Poland, more devastated by warfare, visibly lagged behind the Czech Republic in terms of development (Okólski 1987). This was reflected to some extent by mortality statistics, as by 1950 the registered life expectancy in the Czech Republic already exceeded that of Poland by 5.9 years for males and 5.2 years for females (Bolesławski 1996; Human Mortality Database 2015). Nevertheless, the changes in mortality levels in both countries since 1950 have been quite similar and not so unequivocal as in Western European countries (Fig. 1). We describe these changes by distinguishing three periods.

The first period comprises a rapid increase in life expectancy that lasted in the Czech Republic until 1961 and in Poland until 1966. In the 1950s and early 1960s, a steady increase in life expectancy was registered throughout the European continent, mainly due to a reduction in the still high mortality of infants and children, which was the main epidemiologic challenge just after WWII. In the Czech Republic and particularly in Poland, the progress in life expectancy was mostly due to economic growth and steady improvement in living conditions (Okólski 1985). In these countries, the establishment of egalitarian, public health care systems certainly played a significant role by providing basic services to all social groups in both urban and remote rural areas (McKee and Nolte 2004; Okólski 1985; Rychtaříková 2004). These health care systems responded efficiently to the major needs of the time, principally through an active maternity policy and massive immunization programs.

As this phase of growth lasted longer in Poland than in the Czech Republic, the former managed to catch up with the latter in terms of life expectancy: in 1966, the difference between Czech and Polish life expectancies was as low as 0.5 years for males and 0.9 years for females. In 1961 life expectancy in the Czech Republic was close to the values registered in France; thereafter, the trends between the two countries started to diverge.

In the second period, the Czech Republic saw a decrease in life expectancy that lasted from 1961 until 1969, followed by a very slow increase from 1969 until 1990, while Poland experienced a decrease for males and a very slow increase for females from 1966 until 1991. The progress in life expectancy halted across Europe in the 1960s, when nearly all European countries entered the third stage of the epidemiologic transition (Omran 1971), during which degenerative and man-made diseases became the main threats to population health. From around the mid-1960s and throughout the 1970s, these groups of causes of death were the main impediment to any continuation of the life expectancy increase. Notwithstanding, as early as the 1970s, Western European countries succeeded in applying comprehensive health programs and efficient prevention measures at the individual level, specifically against cardiovascular mortality, traffic accidents and other causes related to risky human behaviour (Vallin and Meslé 2004, 2006), and life expectancy started to increase anew. At the same time, health policies in Central and Eastern Europe did not respond efficiently and flexibly to these emerging epidemiologic threats.

In the countries of Central and Eastern Europe, large population groups exhibited very unfavourable tendencies which, over the following three decades, contributed to a major health crisis (Okólski 1985). Between 1961 and 1990, life expectancy in the Czech Republic decreased by 0.1 years for men and increased by 1.8 years for women (Human Mortality Database 2015). In Poland, the health crisis appeared to be more severe for males, whose life expectancy at birth decreased by 0.8 years (1966–1991), while that of females increased by 2.2 years. As these changes were particularly disadvantageous for males in Poland, the difference in male life expectancy between the two countries under analysis increased anew and in 1991 reached 2.3 years (with a 0.6-year difference in female life expectancy).

The third period marks a steady rebound to a rising trend in life expectancy. The first signs that the end of the health crisis had arrived in Central Europe were observed in the Czech Republic in 1990, when a sustained decrease in death rates began. Soon afterwards, the life expectancy increase recovered in other Central European countries that were undergoing economic and political transitions: Poland and the GDR in 1992, Hungary in 1994, Slovakia in 1996, Romania in 1997 and Bulgaria in 1999. Remarkably, this increase was relatively rapid in the Czech Republic and Poland, and the difference in life expectancy between the two countries has remained stable since 1991 (about 2.2 years among males and 0.2 years among females). In contrast, for the countries of the former USSR, the situation was characterized as a ‘chronic health crisis’ (Meslé 1991), which only recently appears to have been overcome (Grigoriev et al. 2014). In Russia, while a sustained increase in life expectancy was registered until the mid-1960s, the trends in life expectancy since then have been subject to huge fluctuations, with a decline (for men) or stagnation (for women) in the average length of life until 2005.

## Data and Methods of Analysis

A longitudinal analysis of mortality changes requires coherent time series by detailed causes of death. However, due to regular revisions of the ‘International Classification of Diseases and Related Health Problems’ (ICD), single cause-of-death time series are disrupted statistically each time the classification is updated (Janssen and Kunst 2004; Meslé 2006). The transition from the 9th to 10th ICD revision in both the Czech Republic and Poland entailed discontinuities in cardiovascular mortality, and these serve as perfect examples of such disruptions (Fihel 2011; Jasiński et al. 2010; Pechholdová 2011; Wojtyniak et al. 2012; Wróblewska 2009). Moreover, the data by causes of death that was coded in accordance with the same ICD version may not be entirely comparable on an international level, as country-specific coding practices can influence the way in which the underlying cause of death is chosen and recorded (Anderson 2011; Meslé 2006). Therefore, in order to ensure historical and geographical comparability, we used a method that aimed at re-establishing single cause-of-death time series in accordance with the latest ICD revision while also correcting, as far as possible, changes resulting from country-specific coding practices (Meslé and Vallin 1996). This method has been successfully applied to several European countries, including (in alphabetical order) Belarus (Grigoriev et al. 2012), the Czech Republic (Pechholdová 2011; Pechholdová et al. 2011), France (Meslé and Vallin 1996; Vallin and Meslé 1988), Moldova (Penina et al. 2010), Poland (Fihel 2011), Russia (Meslé et al. 1996), Ukraine (Meslé and Vallin 2003, 2012) and West Germany (Pechholdová 2009).

The data applied for the Czech Republic and France were reconstructed across the last three ICD revisions (ICD-8, ICD-9 and ICD-10, see Table 1). The reconstruction for Poland is extensive and also includes ICD-7, as well as estimates of data missing due to the physicians’ strike during the years 1996–2002 (Fihel et al. 2013). For the Czech Republic and France, the transition from the ICD-9 to ICD-10 was carried out in finer detail for causes of death, but only in respect to the disruptions observed for the year when the classification changed. Russia and the former USSR used their own classification system, which made reference to successive revisions of ICD. In this analysis, we use data that were reconstructed for the period 1956–1998 (Meslé et al. 1996) and extended until 2009 in accordance with an abridged version of ICD-10.

**Table 1:** Cause-of-death data used in the analysis for the Czech Republic, France and Poland, in accordance with the ICD revision in use at the time

ICD revision	Czech Republic	France	Poland
ICD-7	–	–	1968–1969
ICD-8	1968–1978	1968–1978	1970–1979
ICD-9	1979–1993	1979–1999	1980–1996
ICD-10	1994–2013	2000–2013	1997–2013

In order to ensure the historical and geographical comparability of mortality trends by causes, we re-established time series by specific causes, first allowing for changes in classifications of diseases and then aggregating them into relatively large groups. In this way, we avoided the problem of insufficient number of deaths in a country with a small population such as that of the Czech Republic. Deaths due to unknown and ill-defined causes were proportionately distributed over deaths due to well-defined causes. The death rates were calculated on the basis of official population estimates, which may raise concerns in the case of Poland that experiences high level of emigration, not accounted for in the national statistics. We elaborate further on this problem in the discussion.

The methods of analysis included comparisons of age-standardized death rates^2^ (ASDRs) by causes of death aggregated into 15 main categories (Table 2), which were grouped according to their similar pathophysiologic or behavioural origins. For instance, causes of death such as alcohol abuse, chronic liver disease, cirrhosis and accidental poisoning by alcohol were grouped into one category of alcohol-related causes. Furthermore, we used the Andreev method for the decomposition of life table changes by age, and by age and causes of death (Andreev 1982; Andreev et al. 2002). Age components of life expectancy changes were computed from 1950, whereas age and cause components were worked out from 1968 because reconstructed data by single cause do not exist for the earlier period for every country under study.

**Table 2:** Causes of death used in the analysis and their codes by ICD-10 category

Causes of death	ICD-10 codes
Infectious diseases	A00-A39; A42-B99; J00-J06; J20-J22
Smoking-related cancers	C00-C14; C32-C34
Stomach cancer	C16
Colorectal cancer	C18-C21
Female breast cancer	C50 (for females)
Prostate cancer	C61
Cervix/uterus cancer	C53-C55
Other cancers	C15; C17; C22-C31; C35-C49; C50 (for males); C51-C52; C56-C60; C62-D48
Alcohol-related causes of death	F10; K70; K73-K74; X45
Diseases of circulatory system	I00-I99
Chronic respiratory disease	J40-J47
Transport accidents	V01-V99
Non-transport accidents	W00-X44; X46-X59; X85-Y98
Suicides	X60-X84
Other causes of death	All other

## Results

### Evolution of Cause-Specific Mortality in the Czech Republic and Poland, Since 1968

#### Infectious Diseases

**Fig. 2:**
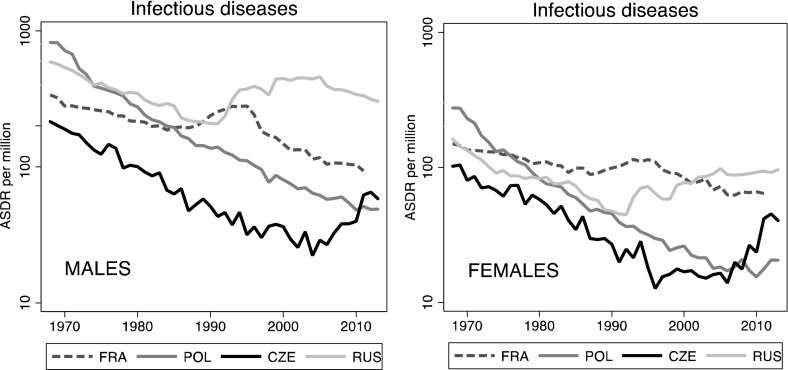
Mortality due to infectious diseases for males and females in four European countries, 1968–2013 (logarithmic scale). *Source*: Own elaboration based on HMD (2015), WHO (2015)

According to the theory of epidemiologic transition, the impact of infectious diseases diminishes as a country develops. In both the Czech Republic and Poland, mortality due to infectious diseases continuously declined until the beginning of the 2000s (Fig. 2). More recently, it started increasing in the Czech Republic, most probably due to better recognition of infectious diseases as underlying causes of death, especially after the introduction of automatic coding in 2011. Contrary to France at the beginning of the 1990s, the Czech Republic and Poland avoided the spread of fatal infections due to the HIV virus because AIDS arrived later in Central European countries, after effective preventive measures were already known and the first effective therapy had been developed. In some ways, the Czech Republic and Poland profited from a certain time lag in the evolution of HIV-related mortality as compared to Western Europe. However, a different situation occurred in Russia, where the death toll from infectious diseases (especially AIDS and tuberculosis) was high in the 1990s and early 2000s.

### Neoplasms Related to Smoking

**Fig. 3:**
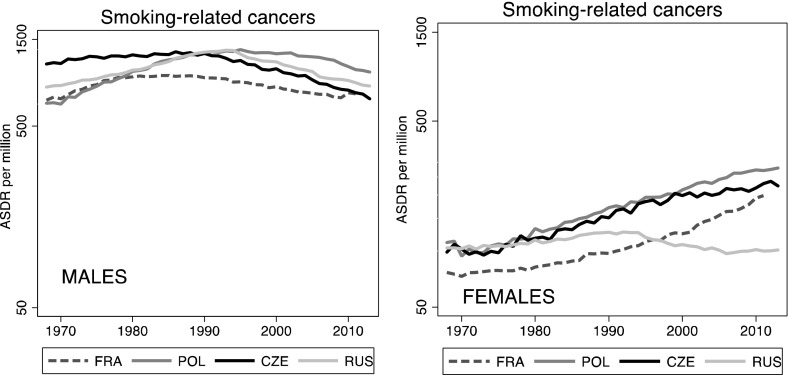
Mortality due to smoking-related cancers for males and females in four European countries, 1968–2013 (logarithmic scale). *Source*: Own elaboration based on HMD (2015), WHO (2015)

Smoking is known to be one of the most important determinants of mortality in European countries (Lopez et al. 1994; Mathers et al. 2009). In all four countries under study, as elsewhere, trends in mortality due to tobacco-related cancers^3^ have been different for males and females (Fig. 3). For the former, a constant increase was observed until the end of the 1980s, followed by a smooth reversal and decrease in the Czech Republic (around 1987), France (1989), Russia (1995) and Poland (1996).

In a quite different development, female mortality due to smoking-related cancers was ten times less than that of males at the end of the 1960s, but was then followed by a steady increase in the Czech Republic, France and Poland. Inversely, although similarly to their male compatriots, mortality from smoking-related cancers has been decreasing for Russian females since the mid-1990s.

### Chronic Respiratory Diseases

**Fig. 4:**
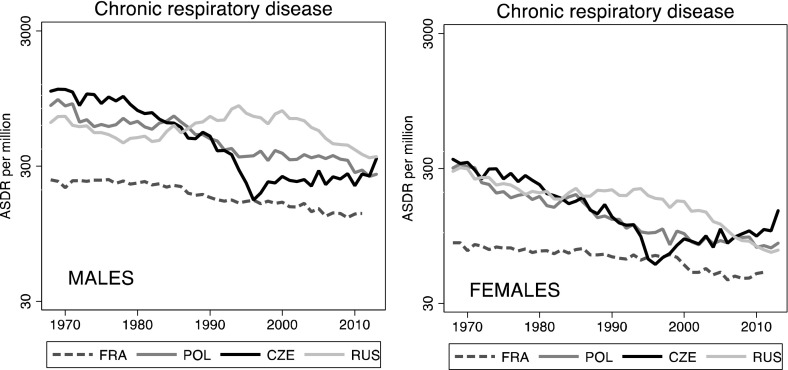
Mortality due to chronic respiratory diseases for males and females in four European countries, 1968–2013 (logarithmic scale). *Source*: Own elaboration based on HMD (2015), WHO (2015)

Among chronic respiratory diseases, the most important cause of death remains chronic obstructive pulmonary disease, which results from tobacco smoking and, to a lesser extent, from occupational hazards. In France and Poland, mortality due to chronic respiratory diseases has been substantially decreasing for both men and women since the end of the 1960s (Fig. 4). In the Czech Republic, the sharp decline registered in the mid-1990s was mostly due to a reduction in mortality from bronchitis, whereas a slow increase was observed thereafter from other chronic airway obstruction diseases. Nevertheless, the general downward tendency in those three countries may be associated with three notable factors: fewer jobs in heavy industry; improvements in working conditions; and a recent drop in smoking prevalence rates. Different observations can be made for Russia, where mortality due to chronic respiratory diseases increased from the mid-1980s until the mid-1990s for females and until the late 1990s for males, then was followed by a decrease.

### Causes of Death Related to Alcohol Consumption

**Fig. 5:**
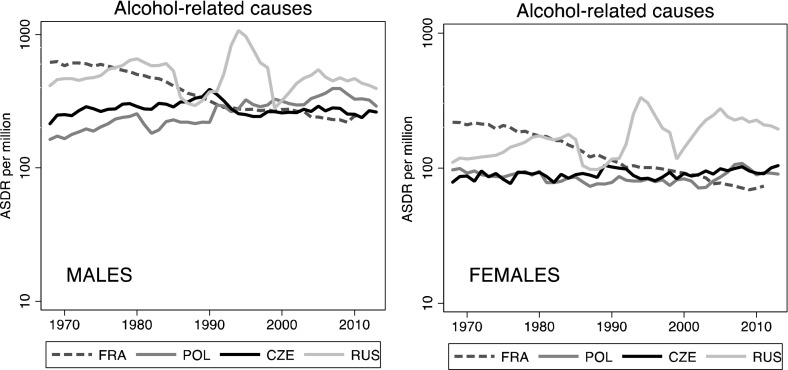
Mortality due to alcohol-related causes of death for males and females in four European countries, 1968–2013 (logarithmic scale). *Source*: Own elaboration based on HMD (2015), WHO (2015)

Between 1970 and 1991, mortality due to alcohol consumption^4^ increased for males in the Czech Republic and stagnated for females in the Czech Republic and Poland (Fig. 5). In Poland, male alcohol-related mortality increased until 1980 and sharply decreased in the period 1980–1982, which can be attributed both to the development of the social movement known as ‘Solidarity’^5^ and to alcohol sales restrictions imposed after the introduction of martial law in 1981. Between 1983 and 1990, male alcohol-related mortality stagnated in Poland. After 1991, different tendencies were registered in both countries: an increase in mortality for Polish males, a decrease for Czech males and stabilization for females in both countries. However, all these trends remained more or less regular in shape, at least as compared to the situation in Russia, which represents a particular pattern of developments in alcohol-related mortality in that it exhibits wide fluctuations.

### Cardiovascular Diseases

Although an analysis by specific cardiovascular diseases would allow us to assess the role of health care systems, life styles and other factors that influence changes in mortality, the comparability of such detailed data does not hold between Poland and the other countries. In Poland, atherosclerosis is reported and coded excessively in relation to other cardiovascular diseases; thus, for the purposes of this study, we aggregated all diseases of the circulatory system into one general category.

**Fig. 6:**
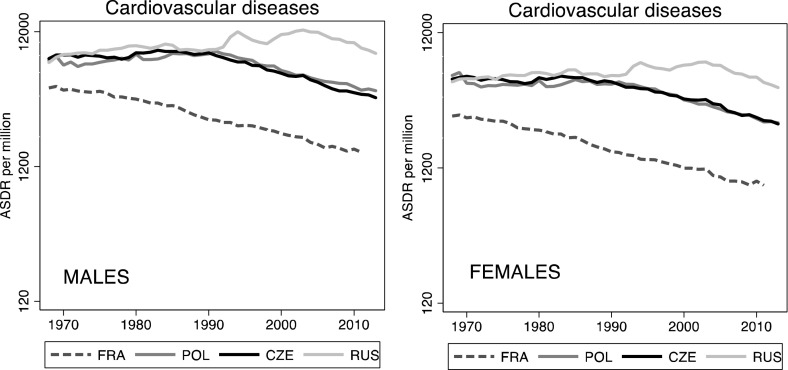
Mortality due to diseases of the circulatory system for men (left) and women (right) in four European countries, 1968–2013 (logarithmic scale). *Source*: Own elaboration based on HMD (2015), WHO (2015)

Between 1970 and the late 1980s, the steady decrease in cardiovascular mortality in France contrasts with a slight increase in the Czech Republic, Poland and Russia (Fig. 6). In the second half of the 1980s, however, mortality from cardiovascular diseases started to decrease for females in the Czech Republic, whereas, in Poland, the trend reversed in the first years of the 1990s. Several factors were recognized as contributing to this shift, and we elaborate on them thoroughly in the discussion. By contrast, the health crisis in Russia persisted and mortality from cardiovascular diseases increased until the early 2000s. It also yielded large variations that are associated with the same risk factors underlying liver cirrhosis, accidents and suicides, such as increased alcohol consumption.

### Accidents, Assaults and Suicides

**Fig. 7:**
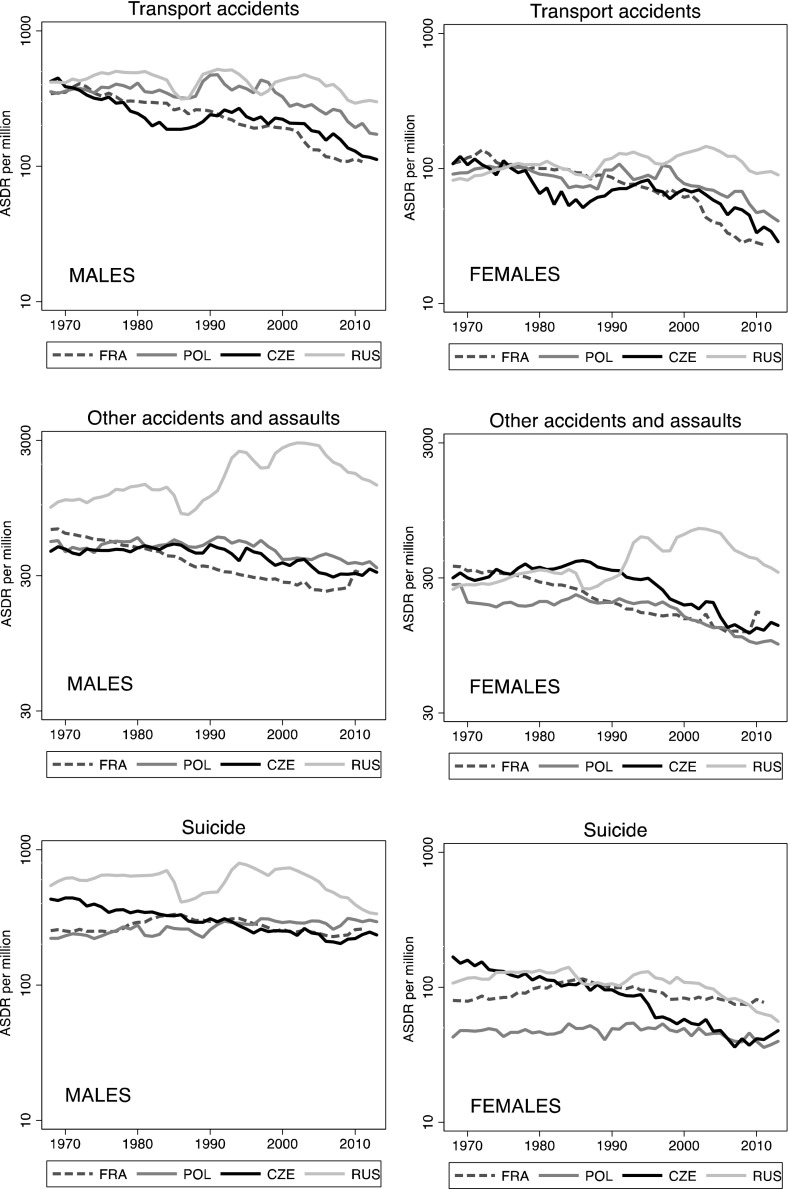
Mortality due to external causes of death for males and females in four European countries, 1968–2013 (logarithmic scale). *Source*: Own elaboration based on HMD (2015), WHO (2015)

Mortality from external causes of death also contributed to a divergence in life expectancies between European countries. While mortality due to transport accidents was gradually diminishing in France, trends for this cause of death fluctuated considerably in the Czech Republic, Poland and Russia, leading to a particularly sharp increase at the turn of the 1980s and into the 1990s (Fig. 7). In the following years, this kind of mortality decreased in the Czech Republic and Poland, but mortality remained elevated and fluctuating in Russia. From the turn of 1980s and 1990s, mortality from other accidents, assaults and suicides diminished in the Czech Republic and in France, remained stable in Poland and exhibited variations in Russia parallel to variations in overall mortality.

### Contribution of Age and Cause Components to the Life Expectancy Changes

Based on the above-presented analysis we distinguished the most important causes of death that contributed to changes in life expectancy in the Czech Republic and Poland. These were infectious diseases, smoking-related cancers, cardiovascular diseases, alcohol-related causes of death and all types of accidents and other external causes. For these causes of death and for distinct periods characterized by stagnation (1968–1991^6^) and improvement (1991 onwards) in life expectancy, we performed an age- and cause-specific decomposition of life expectancy changes.

**Fig. 8:**
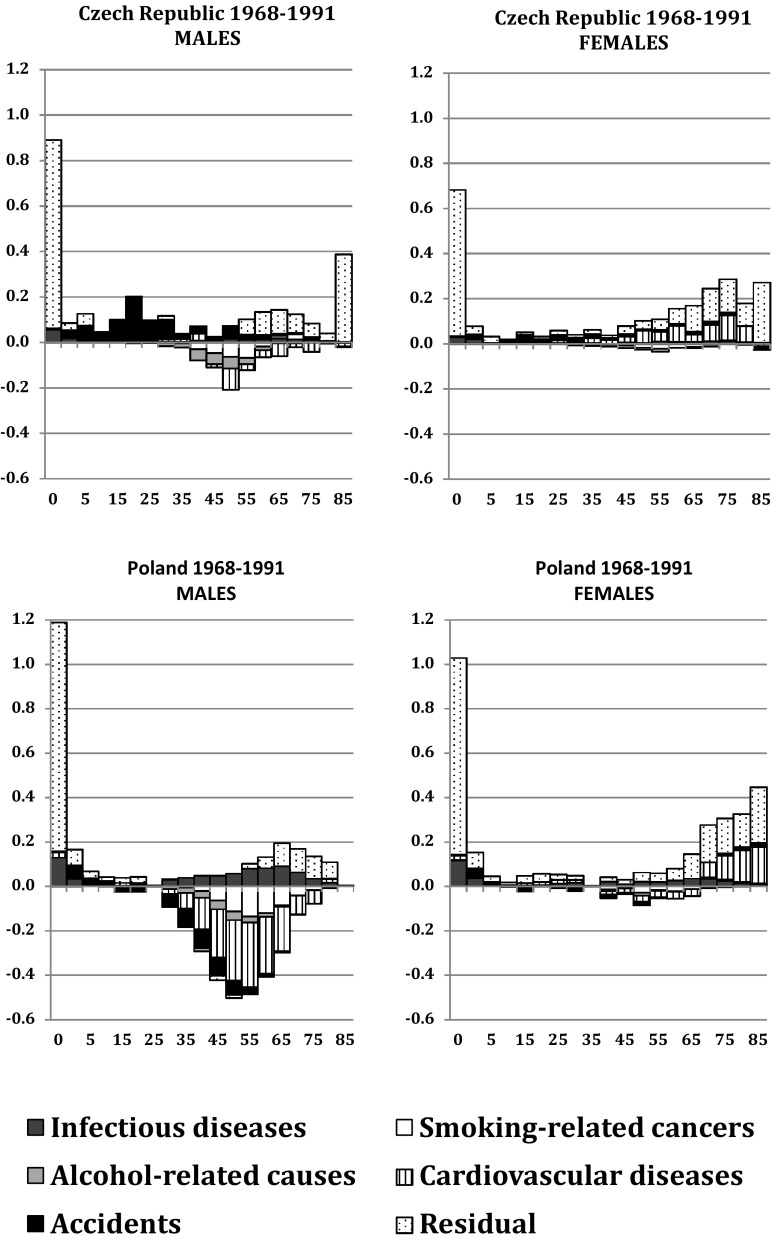
Age and cause components of life expectancy changes by sex (in years) in the Czech Republic and Poland, 1968–1991. *Notes*: To improve the clarity of the figure, causes of death with a less important contribution to life expectancy changes are included in the residual category. This applies to cancers not related to smoking and chronic respiratory disease. It was also necessary to group transport accidents, other accidents, assaults and suicides into one category. *Source*: Own elaboration based on HMD (2015), WHO (2015)

Between 1968 and 1991, in the Czech Republic changes in mortality levels for females at all ages fostered an increase, albeit moderate, in life expectancy (Fig. 8). For males, on the contrary, the decrease in mortality below age 30 contrasted with an increase between the ages of 30 and 59. Similarly to the Czech Republic, Poland’s gain in female life expectancy was due to a decrease in mortality rates at all ages, whereas, for males, unfavourable changes in mortality levels at age 15 and above cancelled out the progress in mortality of infants and children.

Men in the Czech Republic experienced a decrease in mortality from chronic respiratory diseases, transport accidents, stomach cancer, infectious diseases, conditions originating in the perinatal period and congenital malformations. In this way, they contributed positively to life expectancy trends between 1968 and 1991, although circulatory diseases had no impact and smoking-related cancers and causes attributable to alcohol consumption acted negatively. Similar results are found for Polish men. However, in their case, the increase in cardiovascular mortality and–strikingly–smoking-related cancers above the age of 30 had a very important negative impact, which resulted in a decrease in life expectancy despite the reduction in infant mortality and infectious diseases. In the same period, female life expectancy increased slowly in both countries. However, in the Czech Republic the increase was due mostly to a decrease in mortality from circulatory diseases, from conditions originating in the perinatal period and from congenital malformations; in Poland, the drop in infant mortality and from infectious diseases played the most important role, with cardiovascular diseases having a negative contribution below age 70.

**Fig. 9:**
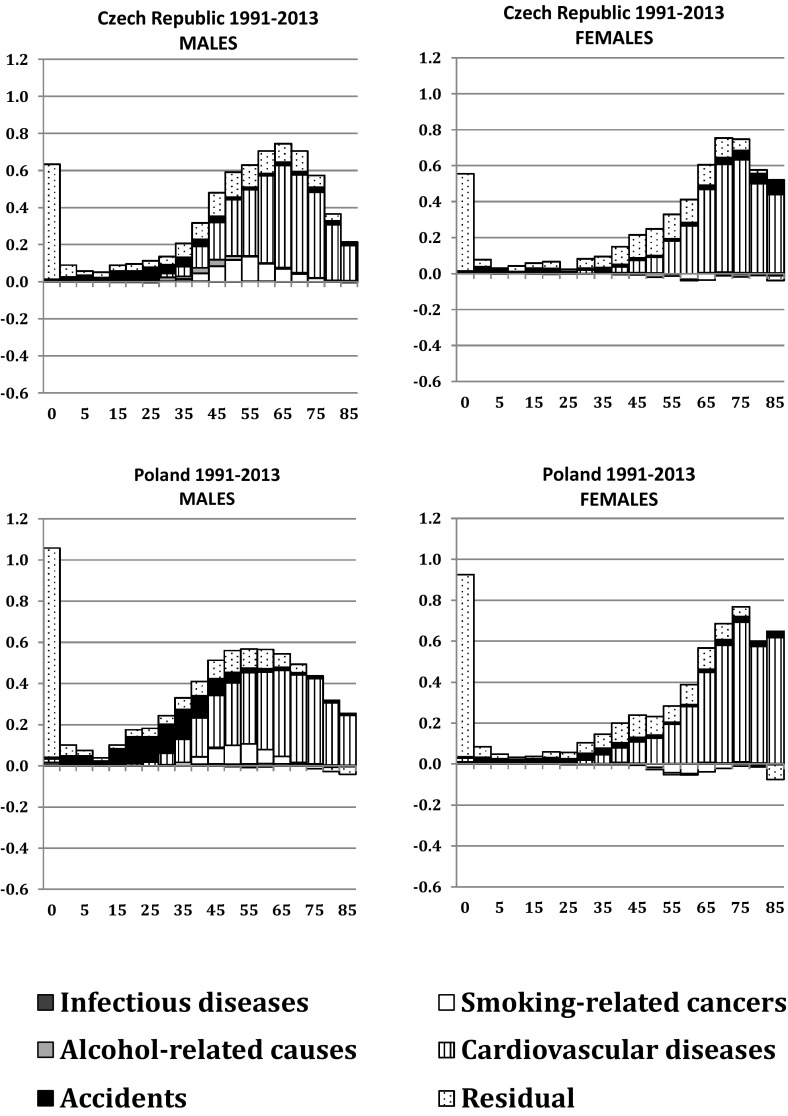
Age and cause components (See notes to Fig. 2.) of life expectancy changes by sex (in years) in the Czech Republic and Poland, 1991–2009. *Source*: Own elaboration based on HMD (2015), WHO (2015)

Between 1991 and 2013, in both the Czech Republic and Poland, the progress in life expectancy trends was stimulated mostly by the decrease in middle- and old-age mortality (Fig. 9). In the Czech Republic, life expectancy increased by 6.9 years for men between 1991 and 2013, with 32% of this change being due to a drop in death rates at ages 30–59 and a further 50% due to a drop at ages 60 and over. Female life expectancy increased by 5.4 years, and the contributions of the drops in death rates in these same age groups amounted to 18 and 67%, respectively, of overall change for women. In Poland, the gain in life expectancy was slightly higher: until 2013, male life expectancy rose by 7.1 years, of which 35% was due to a drop in death rates at ages 30–59 and 38% at ages 60 and above; it rose for women by 5.8 years, of which 15% was due to a drop in death rates at ages 30–59 and 64% at ages 60 and above. In both countries the life expectancy increase was higher for males (0.53 years annually in the Czech Republic and 0.55 in Poland) than for females (0.42 and 0.45, respectively).

These favourable trends were due mostly to the decrease in mortality from perinatal causes for infants and, for adults, from cardiovascular diseases, smoking-related cancers and accidents. For both sexes and in both countries, cardiovascular diseases contributed to more than 50% of the gain in life expectancy. A smaller, albeit still significant, contribution to the increase in life expectancy came from: conditions originating in the perinatal period and congenital malformations (for both sexes in both countries); smoking-related cancers and all types of accidents (for males in both countries); and chronic respiratory diseases (for females in the Czech Republic).

## Conclusions

The Czech Republic and Poland exhibited very similar and almost simultaneous changes in life expectancy trends: a sharp increase until the beginning of the 1960s, driven mostly by a decrease in infant and child mortality; stagnation until the beginning of the 1990s, caused by an increase in adult mortality from circulatory diseases, smoking-related cancers and accidents that counterbalanced the continuing decrease in infant mortality; and a rapid return to the path of sustained growth just after the fall of the Berlin Wall, which was driven mostly by a reduction in middle- and old-age mortality from cardiovascular diseases, smoking-related cancers and traffic accidents. As in other countries of the region, the unfavourable tendencies between the mid-1960s and the end of the 1980s affected men more than women. The situation in Poland was less advantageous than in the Czech Republic, due to increasing male mortality from cardiovascular diseases. In the case of female mortality, however, very similar changes in life expectancy, age- and cause-specific mortality took place in both countries from at least the end of the 1960s. The developments in mortality from circulatory diseases, smoking-related cancers and accidents drove the changes in life expectancy in both countries and in both periods of analysis; however, they contributed to stagnation in the mean duration of life before 1991, and then afterwards to its increase.

## Discussion

### The Leading Causes of Death: Cardiovascular Diseases, Smoking-Related Cancers and Traffic Accidents

Our findings concerning the evolution of cause-specific mortality and determinants of life expectancy changes were in line with other epidemiologic and demographic studies. As for cardiovascular mortality, its above-presented sustained decrease in France corresponds to the application of new technologies and health policies from the 1960s and 1970s (Vallin and Meslé 2006). During the Communist period, the health care systems in the other three countries failed to provide their whole population with specialized skills and medical treatments for cardiovascular and other degenerative diseases, although they were relatively efficient in carrying out large vaccination programs and maternity policies (McKee and Nolte 2004; Rychtaříková 2004). The organization of specialized health care institutions seemed to be too hierarchical, centralized and, in the case of Poland, inadequate for the spatial distribution of the population (Kędelski 1993; Okólski 1985, 1987). There were severe and permanent shortages of physicians, technology and pharmaceuticals, including vitamins, antibiotics, analgesics and modern drugs for treating cardiovascular diseases (McKee and Nolte 2004; Okólski 1985).

Furthermore, the increase in cardiovascular mortality in Central and Eastern Europe had a more complex basis than the structural inefficiency of health care systems. This region seemed to reflect other dysfunctions of the social and economic systems, which contributed to the health crisis (Okólski 1985). A multitude of factors hampered daily activities, such as permanent shortages of food and other basic necessities, the deteriorating quality of food, a poorly diversified diet, bad natural environmental conditions, relatively low wages, poor transportation systems that prolonged commuting times, bad work conditions and the spread of occupational hazards (Bobak and Marmot 1996; Kędelski 1993; Okólski 1985). These difficulties had a major impact on individual well-being, in that they heightened the level of everyday stress and provoked feelings of alienation and of inadequate control over one’s life (Bobak and Marmot 1996; Cockerham 1997; Hanke et al. 1992; Okólski 2004). By extension, these effects went further by spreading self-harming behaviours such as tobacco and alcohol abuse. So long as the living and working conditions failed to improve in the 1990s, the public campaigns continued to be unsuccessful in stigmatizing risky behaviours and preventing the development of circulatory diseases through screening programs.

The substantial decrease in cardiovascular mortality observed in the Czech Republic and Poland since at least 1991 may be attributed to two groups of factors: improvements in the health care systems and treatment methods, and the shift in lifestyles and disease awareness (Burcin and Kucera 2009; Cifkova et al. 2010; Gierlotka et al. 2014; Rychtaříková 2004; Rywik et al. 2003). Although the comparability of data gathered via surveys remains limited in an international context, we elaborate on the prevalence of risk factors, disease awareness and control of cardiovascular diseases in the further parts of the discussion.

As for smoking-related cancers, in the four presented countries we observed an increase and subsequent decrease in male mortality, and an increase (in the Czech Republic, France and Poland) or decrease (in Russia) in female mortality. The results for men should be interpreted as due to the shifts in smoking behaviours, notably a drop in the daily smoking prevalence rates that began in 1980 (Ng et al. 2014). Additionally, this reduction in smoking prevalence rates coincided in Central and Eastern Europe with the replacement of low-quality tobacco by imported cigarettes (Zatoński and Przewoźniak 1999). For men in Russia, other factors were proved to play a significant role: first, the notable attrition of cohorts for whom access to tobacco products was less restricted at young ages (Shkolnikov et al. 1999a), and, second, the increase in mortality from competing causes of deaths, such as cardiovascular diseases and accidents (Shkolnikov et al. 1999b). In turn, increasing female mortality due to smoking-related cancers corresponds to stable (in the Czech Republic and Poland) or increasing (in France) prevalence of female smoking (Ng et al. 2014). In Russia, as female smoking prevalence rates have remained unchanged since 1980 (ibidem), the decrease in mortality has been probably due to factors other than smoking behaviours, notably the above-mentioned cohort effect and competing mortality from other causes of death.

The rise in mortality due to transport accidents in the Czech Republic, Poland and Russia at the turn of the 1980s and 1990s may be attributed to the rising alcohol consumption. The sudden increase in the availability of second-hand Western vehicles, not accompanied by an appropriate development of road infrastructure, also played an important role (Nolte et al. 2000). However, in the Czech Republic and Poland, just like in France in the 1970s, the rising mortality due to transport accidents soon started to decrease thanks to the introduction of road safety controls and regulations such as restricted speed limits and mandatory use of seat belts by all passengers.

Finally, our study showed that the mortality reduction in the Czech Republic and Poland in the 1990s and 2000s was relatively stable, whereas large fluctuations in mortality were observed in Russia, mostly due to infectious diseases, alcohol-related causes and all types of accidents. This evolution has been attributed to a profound social crisis (Shkolnikov et al. 1996; see also Cockerham 1997) in which not only life and work conditions mostly worsened, but also the accessibility and quality of health care services. In this context, government policies appeared ineffective over the long term. For instance, the impact of the anti-alcohol campaign implemented in the mid-1980s (Meslé 2004; Shkolnikov et al. 1996) was brief, and a sudden rise in alcohol consumption related to the deep social crisis pushed the mortality rate even higher than it had been before the anti-alcohol campaign. Regulations on the production and sale of alcohol introduced in 2006 (Neufeld and Rehm 2013) may have a sustained effect on mortality decline, especially due to cardiovascular diseases (Grigoriev et al. 2014), but it is still too early to assess this.

### The Role of Institutional Changes in the Czech Republic and Poland

The reversal of unfavourable mortality trends occurred in the Czech Republic a few years earlier, whereas in Poland it took place simultaneously with reforms to the political and economic system. Institutional changes resulting from the transition to democracy and a market economy, which occurred at the turn of the 1990s, followed a specific course in each of the Central and Eastern European countries. In both the Czech Republic and Poland, the governments in power applied rapid and radical reforms that have been called ‘shock therapy’ (Bałtowski and Miszewski 2006; Koyame-Marsh 2011). These often resulted in transitional recession, hyperinflation, unemployment and a lowering of living conditions in the first years of institutional change. At the beginning of the 1990s, the Czech Republic was already one of the most developed post-Communist countries and, not surprisingly, the political and economic reforms proceeded relatively smoothly. The gross domestic product in this country rapidly returned to a path of sustained growth, while rates of unemployment and of poverty remained relatively low. By contrast, the process of pauperization in Poland seemed to be more severe due to persisting economic inequalities at regional level. For many years, the labour market situation remained difficult, due mostly to numerous bankruptcies and reorganizations of state enterprises, with the rate of unemployment reaching a peak of 20% in 2002. Despite some adverse consequences of the political and economic reforms, however, the institutional changes introduced in both countries resulted in sustainable economic growth and political stability. The Czech and Polish societies repeatedly expressed their support for the pro-Western course of changes, and both became members of the North Atlantic Treaty Organization (in 1999) and of the European Union (in 2004).

The transition to a market economy entailed a decentralization of the health care system and the development of private medical practice. The commercialization of medical services was more advanced in Poland than in the Czech Republic, with the share of expenditures on private medical care accounting for 28–30% of all spending on health in the former and approximately 15% in the latter (OECD 2015). However, while certain authors stress the consequent improvement in admittance to selected medical services due to privatization (Okólski 2004), others mention that this access remained unequal and restricted to patients of higher socio-economic status (Bijak 1999). Yet, in general, the efficiency of the entire health sector improved gradually in both countries due to organizational changes in medical institutions and increases in funding (Bryndova et al. 2009; EOHCS and WHO 1999). In fact, the total health expenditures of both countries rose rapidly in both absolute and relative terms (Table 3 in “Appendix”). Moreover, as members of the European Union, the Czech Republic and Poland started to implement modern health policies that included, for instance, screening programs against certain malignant neoplasms, tobacco-smoking bans in public places and anti-alcohol measures.

**Table 3:** Total expenditures on health in the Czech Republic and Poland, selected years. *Source*: Own elaboration based on OECD (2015)

Country	1990	1995	2000	2005	2010
Total health expenditure per capita, in USD purchasing power parity					
Czech Republic	476.6	794.5	934.5	1425.1	1878.5
Poland	264.7	373.0	562.6	806.3	1335.0
Total health expenditure as % of GDP					
Czech Republic	3.90%	5.90%	6.00%	6.70%	7.20%
Poland	4.40%	5.00%	5.30%	5.80%	6.50%

The number of non-governmental organizations (NGOs) rose steadily in the period of transition, and they contributed to the development of health care services in the domains where state provision was inadequate. While the majority of public expenditures was devoted to current medical practices (EOHCS and WHO 1999), renovations to medical institutions and replacement of equipment were financed by other, non-public resources. For instance, after 1993 the Polish non-governmental organization Great Orchestra of Christmas Charity^7^ donated to public hospitals as many as 30 thousand medical devices^8^ paid for by fund-raising concerts and income tax transfers. Apart from philanthropic activities, many NGOs undertook actions to disseminate knowledge about certain diseases, their determinants and ways of avoiding them while also encouraging sports activities, safe behaviour on the road and quitting smoking.

As a result of institutional changes in the Czech Republic and Poland, modern methods such as coronary angioplasty and surgery, the use of inhibitors, anti-arrhythmic and anti-hyperlipidemic drugs were increasingly applied in the treatment of cardiovascular diseases (Bandosz et al. 2012; Burcin and Kucera 2009; Nolte et al. 2002; Rychtaříková 2002, 2004; Tunstall-Pedoe et al. 2000). The study entitled ‘Monitoring trends and determinants in cardiovascular disease’ (MONICA) showed a significant improvement in the control of hypertension in both populations as compared to the situation in the 1980s (Cifkova et al. 2010; Tykarski et al. 2005). The most recent analysis for Poland proved that the frequency and methods of treatment of patients afflicted by acute myocardial infarction do not differ from practices in countries such as Denmark, Germany or the UK (Gierlotka et al. 2014). Consequently, higher mortality from this disease appears to have resulted from higher prevalence of the risk factors than in other European countries.

### Changes in Health-Related Attitudes and Behaviours

The most important risk factors underlying the development of cardiovascular diseases include diet rich in saturated fat, obesity, hypertension, low physical activity, diabetes, exposure to tobacco, alcohol drinking (Mendis et al. 2011). We discuss the three most important changes that contributed to decreases in mortality from cardiovascular diseases in both countries in the 1990s and 2000s: a reduction in the prevalence of tobacco smoking and shifts in both alcohol consumption and diet. Obviously, these changes did not affect cardiovascular mortality instantaneously, as tobacco, alcohol and unhealthy diet have a lagged and cumulative impact on the development of cardiovascular diseases. However, the authors of the MONICA study registered a significant drop in the coronary risk factor score for both countries under study as early as in the 1980s (Tunstall-Pedoe 2003), so the favourable shifts in people’s behaviours have lasted long enough to have an impact on cardiovascular mortality.

**Fig. 10:**
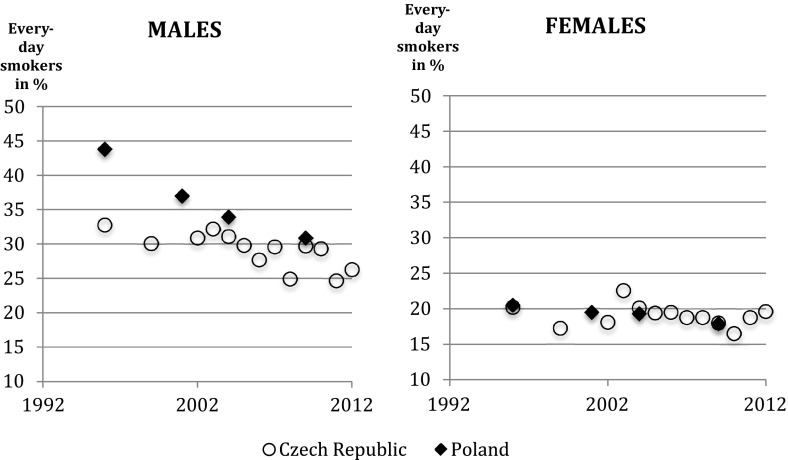
Percentage of adults (aged 15 and over) consuming tobacco every day, 1996–2012. *Source*: Own elaboration based on OECD (2015)

In particular, tobacco became less popular; several epidemiologic surveys (Eurostat 2009; Ng et al. 2014; OECD 2015; Zatoński 2008) have proved that, since the beginning of the 1990s, smoking prevalence rates have been falling in both countries for men and have remained stable for women (Fig. 10 in “Appendix”). In the case of Polish males, the decrease in daily smokers was impressive: from 44% in 1996 to 31% in 2009, which represents a drop of more than 1.5 million persons. This decline is one of the main factors of the recent changes in mortality from cardiovascular diseases (Bandosz et al. 2012; Rywik et al. 2003). The shift in smoking behaviours among Polish males can be attributed to the growing awareness of personal health on the one hand and the influence of organizations that encourage quitting smoking on the other hand. The latter can be attributed mostly to medical authorities, the mass media and NGOs such as the Foundation of Health Promotion (Zatoński 2008). In both countries under study, restrictions on advertising tobacco products and administrative bans on smoking in public places were not introduced until the mid-1990s and early 2000s, but their effects on a decrease in exposure to environmental tobacco smoke has not been insignificant.

**Fig. 11:**
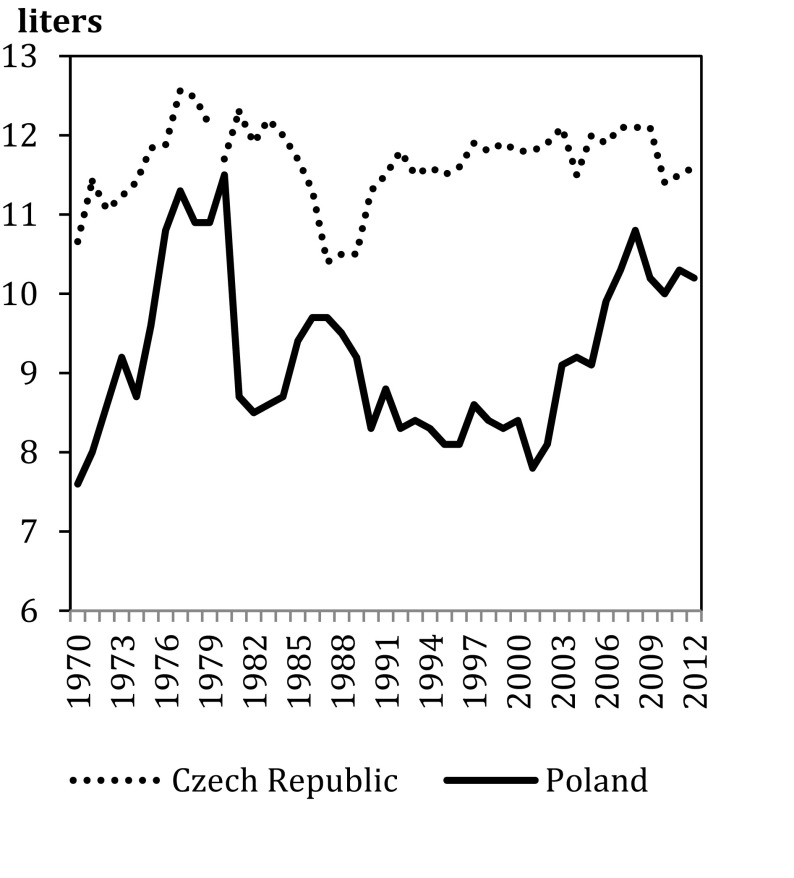
Recorded alcohol consumption (in litres of pure alcohol) per capita (aged 15 and over) in the Czech Republic and Poland, 1970–2012. *Source*: Own elaboration based on OECD (2015)

According to official statistics, alcohol consumption is higher in the Czech Republic than in Poland (Fig. 11 in “Appendix”). However, when allowing for unregistered production in Poland, the level of actual consumption of pure alcohol would appear to be similar in both countries (Kłos 1997; Moskalewicz and Wieczorek 2009). In the beginning of the 1990s, the structure of beverages consumed differed in the Czech Republic and Poland, with the domination of beer in the former (representing around 60% of all alcohol units) and of spirits in the latter (also approximately 60%). During the economic transition, however, the structure of consumed beverages changed radically among the Polish and started to resemble that of the Czechs, with 20% of alcohol units drunk in the form of spirits and 60% of beer (OECD 2015; Zatoński 2008). Although the amount of alcohol consumed in Poland increased in the 2000s (Zatoński et al. 2015), the new structure of beverages seems to have a smaller influence on the morbidity and fatality rate of cardiovascular diseases. Other studies prove that these changes in alcohol consumption in Poland have had a visible impact on mortality due to alcohol by single causes of death: while the importance of chronic diseases (alcoholic liver disease, chronic hepatitis, fibrosis and cirrhosis of liver) steadily increased in the 1990s and 2000s, mortality from other alcohol-related causes (mental and behavioural disorders, accidental poisoning and exposure to alcohol) fluctuated widely and did not exhibit a downward trend (Pechholdová and Fihel 2013).

According to surveys, the share of overweight or obese persons in the Czech Republic and Poland was comparable and slightly increased in the 1990s and 2000s (CSO 2012; OECD 2015; UZIS CR 2011). However, the daily diet became more diversified and richer in fresh fruits and vegetables, especially in Poland, where the consumption of fruit increased by 40% in the period 1991–2011 (FAOSTAT 2015). Also, unsaturated fats started to substitute saturated fats, with an increase of up to 44% in the Czech Republic and 30% in Poland over the period 1993–2011 (FAOSTAT 2015). Many epidemiologic studies stress the role of a more healthy diet in reducing cardiovascular mortality in the Czech Republic and Poland during the economic transition (Nolte et al. 2000; Rychtaříková 2002, 2004; Waśkiewicz et al. 2006; Zatoński 2008; Zatoński et al. 1998; Zatoński and Boyle 1996). For instance, according to Bandosz et al. (2012), lower levels of detrimental types of cholesterol contributed to a 39% decrease in mortality due to coronary heart disease in Poland between 1991 and 2005, and this impact was the most important of all single risk factors for this disease.

### Structural Changes in Societies Under Transition

There is a growing body of research devoted to the socio-economic differences in health, morbidity and mortality in Central and Eastern Europe. As elsewhere, people who are highly educated, married, living in urban areas or have a high socio-economic status are known to live longer and in better health than individuals with different characteristics (Lopez et al. 1995; UN et al. 1982; Valkonen 1993). In Central and Eastern Europe, as in other regions of the continent, mortality was higher and self-assessed health poorer among persons with lower levels of education, and this difference was particularly large in the case of mortality from cardiovascular diseases, alcohol- and smoking-related causes (Mackenbach 2006; Mackenbach et al. 2008). The Czech Republic and Poland are no exceptions to this rule (Jha et al. 2006; Mackenbach 2006; Vandenheede et al. 2013). During the institutional transition of the 1990s and 2000s, the socio-economic discrepancies in mortality widened in some countries such as Estonia, Lithuania and Russia (Jasilionis and Stankuniene 2011; Kalediene and Petrauskiene 2005; Leinsalu et al. 2009; Shkolnikov et al. 1998, 2006). Similarly, in the Czech Republic and Poland, mortality fell for all categories of populations, but inequalities in mortality between the highest and the lowest educational groups increased slightly (Leinsalu et al. 2009; Shkolnikov et al. 2006).

Structural changes in these two countries might have favoured mortality decline, for example, through increases in the share of individuals with a tertiary level of education or in those who are employed in the services sector and living in urban or suburban areas. Due to their better economic status, it is presumed that university graduates have wider access to health care and are more able to withstand the adverse consequences of political and economic transition (Shkolnikov et al. 2006). What is more, they more frequently lead healthy lifestyles and are more aware of determinants and symptoms of diseases than people in other educational categories. Consequently, an increase in the proportion of highly educated persons may result in an improvement in the average length of life. Indeed, according to population censuses, the proportion of adults with a tertiary level of education increased in the Czech Republic from 7.2% in 1991 to 14.8% in 2011, and in Poland from 6.5% in 1988 to 17.4% in 2011. Shkolnikov et al. (2006) proved that changes in the population educational structure in the Czech Republic contributed to an 11% increase in life expectancy at birth for men and 7% for women between 1984–1985 and 1999–2000. Due to the lack of data concerning census-linked death counts, this effect has not been calculated for Poland. However, the spread of education may have had a similar impact on life expectancy because both countries experienced comparable changes in the structure of education.

### Data Limitations

This study encountered two important methodological problems when using the data on causes of death. First, due to relatively frequent registration of deaths from unknown, ill-defined or so-called garbage causes, the mortality data for Poland are evaluated as being of low quality (Mathers et al. 2005). Some studies have shown that a high number of deaths were registered as due to unknown and ill-defined causes because, when people died at home, physicians coding the underlying cause of death very often had inadequate knowledge of the medical documentation of disease (Fihel et al. 2014; Jędrychowski et al. 2001). As other regional studies have shown that unknown and ill-defined codes used to be assigned in place of diabetes (Bijak 2003), we applied the Ledermann method (Ledermann 1955) and tested the correlation between the frequency of assignment of ill-defined causes (‘R’ codes) and specific well-defined causes at the regional level. We found statistically significant negative correlations for the following causes: acute myocardial infarction, heart failure (a ‘garbage’ code itself), pneumonia and non-insulin-dependent diabetes mellitus. This means that these causes of death are probably assigned instead of ill-defined causes, and vice versa. This finding requires additional investigation in forthcoming analyses.

The second issue concerns the reliability of population estimates used for calculating death rates for Poland. For four decades now, Poland has been experiencing several waves of emigration: after the wave of political repression in 1981 (Sakson 2002); due to the liberalization of border traffic in the 1990s (Iglicka and Sword 1999); and following Poland’s 2004 entry into the European Union (Black et al. 2010). Most emigrants left Poland without deregistering from their place of permanent residence and are still included in the official estimates of the overall population. However, the 2011 population census revealed that Poland’s stock of permanent residents living abroad for at least 1 year was as high as 1.565 million (CSO 2013). Such intensive outflow was not observed in the Czech Republic, for which the population estimates remain reliable.

**Fig. 12:**
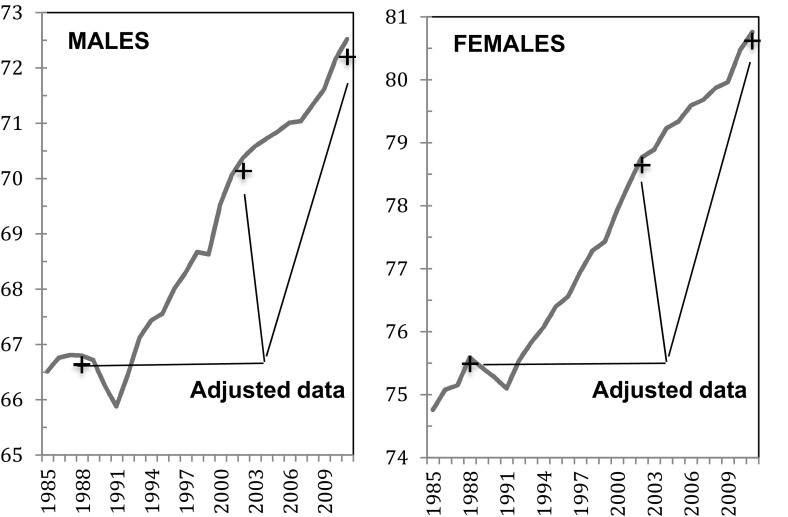
Male and female life expectancy at birth (in years) in Poland according to the HMD (2015) and adjustments allowing for unregistered emigration, 1985–2011. *Source*: Own elaboration based on the HMD (2015), CSO (2003, 2013), Sakson (2002)

In order to assess the impact of unregistered outflows on demographic rates in Poland, crude, cause- and age-specific death rates were recalculated by allowing for population losses due to unregistered emigration. This was possible only for years of population censuses showing the exact age structure of emigrants, i.e. 1988, 2002 and 2011. Next, we calculated standardized cause-specific and overall death rates, as well as life expectancies at birth. Not surprisingly, the highest impact of unregistered outflow is associated with two factors: the period following the 2011 Polish accession into the European Union, when emigration was the most intensive, and for causes of death such as transport accidents and suicides that occur at the most mobile ages, that is in their 20s and 30s. However, the weight of these causes of death for overall mortality is relatively small, and the consequent change in overall death rates and life expectancies appears to be marginal. For instance, the male population loss of 9% at ages 25–29 in 2011 translated into a 4% increase in the age-standardized death rate due to transport accidents. More generally, the population loss of men at all ages in 2011 entailed a 1.7% increase in the age-standardized death rate and a 0.31-year decrease in life expectancy at birth (Fig. 12 in “Appendix”). It appears that unregistered outflow from Poland, though large in scale, involves mostly young persons for whom the level of mortality is too low to have a substantial effect on Polish life expectancy. Consequently, our conclusions on the cause-of-death determinants of life expectancy changes based on the population estimates remain quite realistic.

## Appendix

See Table 3 and Figs. 10, 11, 12.

## Footnotes

1 Not counting the case of the German Democratic Republic, which had a very specific course of changes related to reunification.

2 We performed a direct standardization based on the European standard population.

3 The following malignant neoplasms were included: lip, oral cavity, pharynx, larynx, trachea, bronchus and lung (ICD-10 codes C00-C14, C32-C34).

4 The following causes of death were included: mental and behavioural disorders due to use of alcohol (ICD-10 code F10), alcoholic liver disease (K70), chronic hepatitis (K73), fibrosis and cirrhosis of liver (K74), accidental poisoning by and exposure to alcohol (X45).

5 In August 1980, the Communist regime allowed for the establishment of autonomous trade unions, but these freedoms were curtailed in December 1981 when martial law and political repression were introduced. This period is popularly called, not without justification, a carnival of Solidarity. Local units of Solidarity were established all over the country, with large-scale participation in meetings, demonstrations and Catholic masses. As censorship had been reduced to some extent, the social atmosphere—if one can call it that—became more optimistic.

6 We present the period starting only in 1968, as no earlier data are available.

7
*Wielka Orkiestra Świątecznej Pomocy.*

8 Tomographs, ultrasonic devices, incubators for neonates, insulin pumps and other equipment for paediatric and geriatric medical units.
